# Age-standardized mortality rates related to viral hepatitis in Brazil

**DOI:** 10.1186/s12879-017-2619-y

**Published:** 2017-07-31

**Authors:** Hugo Perazzo, Antonio G Pacheco, Paula M Luz, Rodolfo Castro, Chris Hyde, Juliana Fittipaldi, Caroline Rigolon, Sandra W Cardoso, Beatriz Grinsztejn, Valdiléa G Veloso

**Affiliations:** 1 Fundação Oswaldo Cruz (FIOCRUZ), Instituto Nacional de Infectologia Evandro Chagas (INI), Laboratório de Pesquisa Clínica em DST e AIDS (LAPCLIN-AIDS), Rio de Janeiro, Brazil; 2 Fundação Oswaldo Cruz (FIOCRUZ), Programa de Computação Científica (PROCC), Rio de Janeiro, Brazil; 3 Institute of Health Research, Peninsula Technology Assessment Group (PenTAG), Evidence Synthesis and Modelling for Health Improvement (ESMI), University of Exeter Medical School, University of Exeter, England, UK

**Keywords:** Liver disease, Hepatitis, Death rates, Age-standardized mortality

## Abstract

**Background:**

Liver-related mortality has been increasing worldwide. We aimed to estimate the age-standardized mortality rates from viral hepatitis in Brazil.

**Methods:**

The Brazilian National Death Registry was analyzed from 2008 to 2014. Viral hepatitis deaths were defined by the following ICD-10 codes in the death certificate: hepatitis A [B15.0; B15.9]; hepatitis B [B16.2; B16.9; B18.1]; hepatitis C [B17.1; B18.2]; hepatitis Delta [B16.0; B16.1; B18.0; B17.0] and other viral hepatitis [B17.2; B17.8; B18.8; B18.9; B19.0; B19.9]. Crude mortality rates were calculated by the ratio between total number of deaths and estimated population. Mortality rates were age-standardized by the direct method using the WHO standard population.

**Results:**

Thirty four thousand ,nine hundred seventy eight deaths had viral hepatitis mentioned in their death certificate [65% male, aged 58 years, 73% associated with hepatitis C]. Age-standardized mortality rate (95% CI) due to viral hepatitis was 2.695 (2.667–2.724) deaths per 100,000 inhabitants: South region had the higher rates [3.997 (3.911–4.085)]. Mortality rates associated with hepatitis A and Delta were 0.032 (0.029–0.035) and 0.028 (0.025–0.031), respectively. Hepatitis C mortality rates were 4-fold higher than those associated with hepatitis B [1.964 (1.940–1.989) vs 0.500 (0.488–0.512)]. South region had the higher rates for hepatitis C [3.163 (3.087–3.241)] and North had the higher rates for hepatitis A [0.066 (0.049–0.087)], B [0.986 (0.918–1.058)] and Delta [0.220 (0.190–0.253)].

**Conclusion:**

Viral hepatitis remains a major public health issue in Brazil. Mortality rates were not homogeneous across the country, suggesting that health policies should be customized according to geographical location.

**Electronic supplementary material:**

The online version of this article (doi:10.1186/s12879-017-2619-y) contains supplementary material, which is available to authorized users.

## Background

In the last years, liver-related mortality rates have been increasing worldwide [1]. Approximately 2 million cases of chronic liver diseases are expected in the next 40 years in Latin America [2]. Viral hepatitis might lead to fulminant liver failure and remains the main indication of liver transplantation due to acute or chronic liver disease [3]. Acute hepatitis A is generally self-limited and more severe disease occurs more frequently in elderly and individuals with immunodeficiency [4]. Hepatitis E infection causes large outbreaks and high mortality rates have been described in pregnant women [5]. Chronic infection by hepatitis B, C and Delta virus leads to progression from minimal fibrosis to cirrhosis, the most frequent liver related death cause worldwide [1].

In Brazil, more than 130,000 new cases of acute and chronic viral hepatitis were registered by the health authorities from 2012–2015 (*Sistema de Informação de Agravos de Notificação* – SINAN – website: http://portalsinan.saude.gov.br/hepatites-virais) [6]. Data from the Ministry of Health (MoH) of Brazil estimated that 1.4–1.7 million people are chronically infected with hepatitis C virus (HCV) [7]. Presence of advanced fibrosis was reported in up to 30% of patients with hepatitis B and Delta coinfection in western Amazon Basin [8, 9] and more than 800,000 liver-related hospitalizations (~30,000 admissions per year) were performed in the last decade in Brazil [10]. We previously reported a nationwide analysis describing an increase in number of deaths due to liver diseases and cirrhosis in the last decade [11]. The Viral Hepatitis National Program of the Brazilian MoH has been implementing strategies to prevent, early-diagnosis and treatment of viral hepatitis [12]. However, the burden of viral hepatitis might not be homogeneous across the country. Thus, the analysis of the geographical distribution the mortality rates related to viral hepatitis seems to be essential to prioritize or reinforce these health policies in hot spots of specific etiologies of viral hepatitis. The aim of this study was to estimate the age-standardized mortality rates related to viral hepatitis in Brazil and its micro and macro regions.

## Methods

### Study design

We analyzed the Brazilian National Death Registry database (*Sistema de Informação sobre Mortalidade -* SIM) from the Brazilian Unified Health System Information Technology Department (DATASUS) to estimate the mortality rates related to viral hepatitis in a 7-year period (from 2008 to 2014). The study protocol was approved with waiver of informed consent by the Ethical Committee from Instituto Nacional de Infectologia Evandro Chagas (IRB 51736815.3.0000.5262). Federative Units and macro-regions of Brazil were defined by the Brazilian Institute of Geography and Statistics (IBGE) as the Brazilian states and legally valid subnational units as North, Northeast, Central-West, Southeast and South regions, respectively.

### Mortality and population data collection

The Brazilian MoH has an open-source and web available database, the Brazilian Death Registry (SIM), where all deaths causes have been registered based on the death certificate (DC) in Brazil since 1979. Anonymous data have been published in a public domain (http://datasus.saude.gov.br/informacoes-de-saude/tabnet/estatisticas-vitais). This database contains demographic characteristics, geographic location and causes of death that occurred in Brazil in the last decades. Primary, secondary and contributing causes of death have been classified according to the 10th revision of the International Classification of Diseases (ICD-10) since 1999. The underlying cause of death is determined as the "disease of injury which initiates the train of events leading directly to death". When more than one cause or condition is listed in the DC, the underlying cause is determined by the sequence of conditions using standard algorithms provided by the Brazilian MoH [13]. Population estimation in Brazil has been provided by IBGE. Socio-demographics’ statistics of individuals living in Brazil have been based on the 10-year interval census (1980, 1991, 2000 and 2010) and the annual inter-census projections performed by IBGE. Population estimates are available in a web-site (http://ibge.gov.br) stratified by age, gender and geographic localization.

### Definition of mortality associated with viral hepatitis

Deaths related to viral hepatitis were defined by the following ICD-10 codes: hepatitis A [B15.0; B15.9] hepatitis B [B16.2; B16.9; B18.1]; hepatitis C [B17.1; B18.2] and hepatitis Delta [B16.0; B16.1; B18.0; B17.0] other viral hepatitis [B17.2; B17.8; B18.8; B18.9; B19.0; B19.9]. A death due to viral hepatitis was defined by the present of any of the ICD-10 codes described above in any field of the DC (primary, secondary or underlying causes of death). Deaths due to viral coinfection were considered by the presence of two or more codes related to different viral hepatitis infections in the DC. An exception was hepatitis Delta, formally known as hepatitis B and Delta coinfection, which was analyzed separately from hepatitis B.

### Mortality rates calculation

Mortality rates due to viral hepatitis in Brazil were analyzed in a 7-year period: from 2008 (after an improvement in quality in cause-of-death information) to 2014 (last year available at the time of the analysis for this paper). Crude and age-standardized mortality rates, expressed per 100,000 inhabitants, were calculated for Brazil and its federative units and macro-regions. The crude mortality rate was calculated as the ratio between total number of deaths due to hepatitis and estimated population. The age-standardized mortality rate was calculated using the direct standardization method using a standard population [14]. The World Population Standard (2000–2025) reported by the World Health Organization (WHO) was used as the standard population [15]. The WHO Population Standard was especially defined to reflect the average age structure of the world’s population expected from the year 2000–2025. The statistical software R (R Foundation for Statistical Computing, Vienna, Austria. URL http://www.R-project.org/) and STATA (StataCorp LP, College Station, TX, USA) were used for data management, calculation of mortality rates and its 95% confidence intervals (CI). Time trends were tested using Poisson regression models with mortality rates as the dependent variable and calendar year as a numerical explanatory variable.

## Results

A total of 8,106,219 deaths were registered in Brazil from January 2008 to December 2014. In this period 0.43% (*n* = 34,978) of deaths had ICD-10 codes related to viral hepatitis mentioned in primary, secondary or contributing causes of death of their DC. Hepatitis C was the most prevalent (73%) etiology in individuals who had viral hepatitis as cause of death in this period. Presence of viral hepatitis co-infection in the DC was rare (<2%). Most individuals who had viral hepatitis as cause of death were male (65%), aged [median (IQR)] of 58 (49–67) years-old and self-declared as Caucasian (61%). Deaths related to viral hepatitis occurred mostly in the Southeast region (52%) which is the most populated region in Brazil (Table 1 and Additional file 1: Figure S1).

**Table 1:** Demographic characteristics of people who had mention of viral hepatitis in their death certificate from 2008 to 2014

	Hepatitis A (*n* = 391)	Hepatitis B (*n* = 5992)	Hepatitis C (*n* = 24841)	Hepatitis Delta (*n* = 353)	Other Hepatitis (*n* = 2859)	All (*n* = 34436)
Male gender; n (%)	218 (55.8)	4516 (75.4)	15,730 (63.3)	240 (68.0)	1657 (58.0)	22361 (64.9)
Age; median (IQR)	52 (24–69)	56 (46–67)	58 (51–67)	45 (35–57)	53 (39–67)	58 (49–67)
Age groups; n (%)						
< 20 years	79 (20.3)	77 (1.3)	27 (0.1)	13 (3.7)	245 (8.6)	441 (1.3)
20–29 years	36 (9.2)	222 (3.7)	201 (0.8)	42 (11.9)	200 (7.0)	701 (2.0)
30–39 years	33 (8.5)	599 (10.0)	1216 (4.9)	75 (21.3)	289 (10.1)	2212 (6.4)
40–49 years	40 (10.3)	1098 (18.3)	4088 (16.5)	84 (23.8)	478 (16.7)	5788 (16.8)
50–59 years	47 (12.0)	1568 (26.2)	7733 (31.1)	62 (17.6)	546 (19.1)	9956 (28.9)
60–69 years	64 (16.4)	1231 (20.6)	6388 (25.7)	35 (9.9)	510 (17.9)	8228 (23.9)
70–79 years	41 (10.5)	830 (13.9)	3723 (15.0)	28 (7.9)	374 (13.1)	4996 (14.5)
> = 80 years	50 (12.8)	364 (6.1)	1456 (5.9)	14 (3.9)	213 (7.5)	2097 (6.2)
Ethnicity; n (%)						
Caucasian	159 (40.7)	3080 (51.4)	16,351 (65.8)	97 (27.5)	1377 (48.2)	21,064 (61.2)
miscegination “*pardo*”	175 (44.8)	1999 (33.4)	5430 (21.9)	197 (55.8)	1049 (36.7)	8850 (25.7)
African-Black	31 (7.9)	499 (8.3)	1712 (6.9)	20 (5.7)	234 (8.2)	2496 (7.2)
Asiatic/Indigenous	6 (1.5)	114 (1.9)	177 (0.7)	14 (4.0)	34 (1.2)	345 (1.0)
Missing	20 (5.1)	300 (5.0)	1171 (4.7)	25 (7.0)	165 (5.7)	1681 (4.9)
Education in years of study; n (%)						
< 1 year	61 (15.6)	518 (8.7)	912 (3.7)	44 (12.5)	303 (10.6)	1838 (5.3)
1 to 3 years	85 (21.7)	1235 (20.6)	4210 (17.0)	81 (23.0)	601 (21.0)	6212 (18.0)
4 to 7 years	79 (20.2)	1330 (22.2)	5479 (22.1)	91 (25.8)	603 (21.1)	7582 (22.0)
8 to 11 years	48 (12.3)	955 (15.9)	4837 (19.5)	52 (14.7)	408 (14.3)	6300 (18.3)
≥ 12 years	21 (5.4)	480 (8.0)	2486 (10.0)	13 (3.7)	194 (6.8)	3194 (9.4)
Missing	97 (24.8)	1474 (24.6)	6917 (27.7)	72 (20.3)	750 (26.2)	9310 (27.0)
Geographic macro-regions; n (%)						
North	58 (14.9)	778 (13.0)	1010 (4.1)	198 (56.1)	391 (13.7)	2435 (7.1)
Northeast	137 (35.0)	872 (14.6)	2448 (9.9)	29 (8.2)	609 (21.3)	4095 (11.9)
Southeast	128 (32.7)	2619 (43.7)	13,841 (55.7)	76 (21.5)	1209 (42.3)	17873 (51.9)
South	41 (10.5)	1170 (19.5)	6448 (26.0)	37 (10.5)	469 (16.4)	8165 (23.7)
Central-West	27 (6.9)	553 (9.2)	1094 (4.4)	13 (3.7)	181 (6.3)	1868 (5.4)

Viral hepatitis were classified according to the following ICD-10 codes: Hepatitis A [B15.0; B15.9] Hepatitis B [B16.2; B16.9; B18.1]; Hepatitis C [B17.1; B18.2] and Hepatitis Delta [B16.0; B16.1; B18.0; B17.0] other viral hepatitis [B17.2; B17.8; B18.8; B18.9; B19.0; B19.9]. This analysis considered only people who have only one viral hepatitis ICD-10 code in their DC (mono-infected individuals). Presence of co-infection (*n* = 552): HAV/HBV (*n* = 13); HAV/HCV (*n* = 7); HBV/HCV (*n* = 518); HCV/HDV (*n* = 8); HAV/HBV/HCV (*n* = 3); HBV/HCV/others viral hepatitis (*n* = 3). Total of deaths = 34,988. Missing: gender (*n* = 3), age (*n* = 10). Mean population per year (in millions inhabitants): North =16,145; Northeast = 54,173; Southeast = 81,931; South = 27,956; Central-West = 14.360 and Brazil = 194,567

Age-standardized mortality rate (95% CI) due to viral hepatitis was 2.695 (2.667–2.724) deaths per 100,000 inhabitants in Brazil in this 7-year period (2008–2014). The distribution of these death rates was not uniform across the country: South region [3.997 (3.911–4.085)] had the higher and the Northeast region [1.261 (1.223–1.301)] the lower age-standardized mortality rates due to viral hepatitis. Mortality rates due to hepatitis C were 4-fold higher than those associated with hepatitis B in Brazil [1.964 (1.940–1.989) vs 0.500 (0.488–0.512)]. Age-standardized mortality rates due to hepatitis A (0.066 [0.049–0.087]), hepatitis B [0.986 (0.918–1.058)] and hepatitis Delta [0.220 (0.190–0.253)] were higher in the Brazilian North region compared to others regions. South [3.163 (3.087–3.241)] and Southeast [2.367 (2.328–2.406)] were the top ranking regions for age-standardized mortality rates associated with hepatitis C. Table 2 summarizes the number of deaths and crude / age-standardized mortality rates due to viral hepatitis according to macro-regions in Brazil from 2008 to 2014. In overall, the mortality rates related to viral hepatitis have decreased in 5.5% from 2008 to 2014 [2.764 (2.685–2.844) to 2.612 (2.542–2.684) per 100,000 inhabitants]. However, there was a non-significant trend (*p* value >0.05) for annual increasing or decreasing mortality rates in all Brazilian macro-regions from 2008 to 2014. Additional file 1 Table S1 describes the annual percent change after age-standardization of mortality rates related to each type viral hepatitis according to the Brazilian macro-regions.

**Table 2:** Number of deaths, crude and age-standardized mortality rates (per 100,000 inhabitants) [95% confidence interval] due to viral hepatitis according to the macro-regions in Brazil from 2008 to 2014

Region	Hepatitis A	Hepatitis B	Hepatitis C	Hepatitis Delta	Other hepatitis	All
North						
Deaths (n)	59	842	1071	212	396	2492
Crude MR	0.052	0.745	0.948	0.196	0.350	2.205
Age-st-MR [95% CI]	0.066	0.986	1.458	0.220	0.449	3.062
	[0.049–0.087]	[0.918–1.058]	[1.370–1.550]	[0.190–0.253]	[0.403–0.498]	[2.938–3.189]
Northeast						
Deaths (n)	141	930	2506	30	617	4153
Crude MR	0.037	0.245	0.661	0.008	0.163	1.095
Age-st-MR [95% CI]	0.039 [0.036–0.046]	0.278 [0.261–0.297]	0.780 [0.750–0.812]	0.009 [0.006–0.013]	0.176 [0.163–0.191]	1.261 [1.223–1.301]
Southeast						
Deaths (n)	139	2900	14,111	78	1226	18,146
Crude MR	0.024	0.506	2.460	0.014	0.214	3.164
Age-st-MR [95% CI]	0.024	0.486	2.367	0.013	0.207	3.045
	[0.020–0.028]	[0.468–0.504]	[2.328–2.406]	[0.010–0.016]	[0.195–0.219]	[3.001–3.090]
South						
Deaths (n)	46	1285	6564	40	471	8281
Crude MR	0.024	0.657	3.354	0.020	0.241	4.231
Age-st- MR [95% CI]	0.023	0.621	3.163	0.019	0.230	3.997
	[0.017–0.031]	[0.588–0.657]	[3.087–3.241]	[0.014–0.026]	[0.210–0.252]	[3.911–4.085]
Central-West						
Deaths (n)	31	592	1131	13	187	1906
Crude MR	0.031	0.589	1.125	0.016	0.186	1.896
Age-st-MR [95% CI]	0.035	0.662	1.284	0.018	0.208	2.151
	[0.024–0.050]	[0.609–0.718]	[1.209–1.362]	[0.009–0.031]	[0.179–0.241]	[2.054–2.251]
Brazil						
Deaths (n)	416	6549	25,383	373	2897	34,978
Crude MR	0.031	0.481	1.864	0.028	0.213	2.568
Age-st-MR [95% CI]	0.032	0.500	1.964	0.028	0.220	2.695
	[0.029–0.035]	[0.488–0.512]	[1.940–1.989]	[0.025–0.031]	[0.212–0.229]	[2.667–2.724]

Mean population per year (ratio between total population from 2008 to 2014 and 7 years; expressed in millions inhabitants): North =16,145; Northeast = 54,173; Southeast = 81,931; South = 27,956; Central-West = 14.360 and Brazil = 194.567. Crude mortality rate = number of death / mean population * 7 (number of years). Age-st-MR, age-standardized mortality rate; MR, mortality rate. *N* = 552 patients had co-infection (more than one viral hepatitis ICD-10 codes in their death certificate: HAV/HBV (*n* = 13); HAV/HCV (*n* = 7); HBV/HCV (*n* = 518); HCV/HDV (*n* = 8); HAV/HBV/HCV (*n* = 3); HBV/HCV/others viral hepatitis (*n* = 3). In patients with missing age (*n* = 10), it was not possible to calculate adjusted-mortality rate

**Fig. 1:**
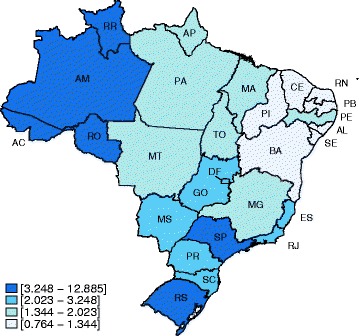
Age-standardized mortality rates (per 100,000 inhabitants) associated with viral hepatitis from 2008 to 2014 in Brazilian federative units. [AC, Acre; AL, Alagoas; AM, Amazonas; AP, Amapa; BA, Bahia; CE, Ceara; DF, Distrito Federal; ES, Espirito Santo; GO, Goiânia; Ma, Maranhão; MG, Minas Gerais; MS, Mato Grosso do Sul; PA, Para; PB, Paraiba; PE, Pernambuco; PI, Piaui; PR, Parana; RJ, Rio de Janeiro; RN, Rio Grande do Norte; RO, Rondônia; RR, Roraima; RS, Rio Grande do Sul; SC, Santa Catarina; SE, Sergipe; SP, São Paulo; TO, Tocantins]. Density of colors expressed in quartiles

**Fig. 2:**
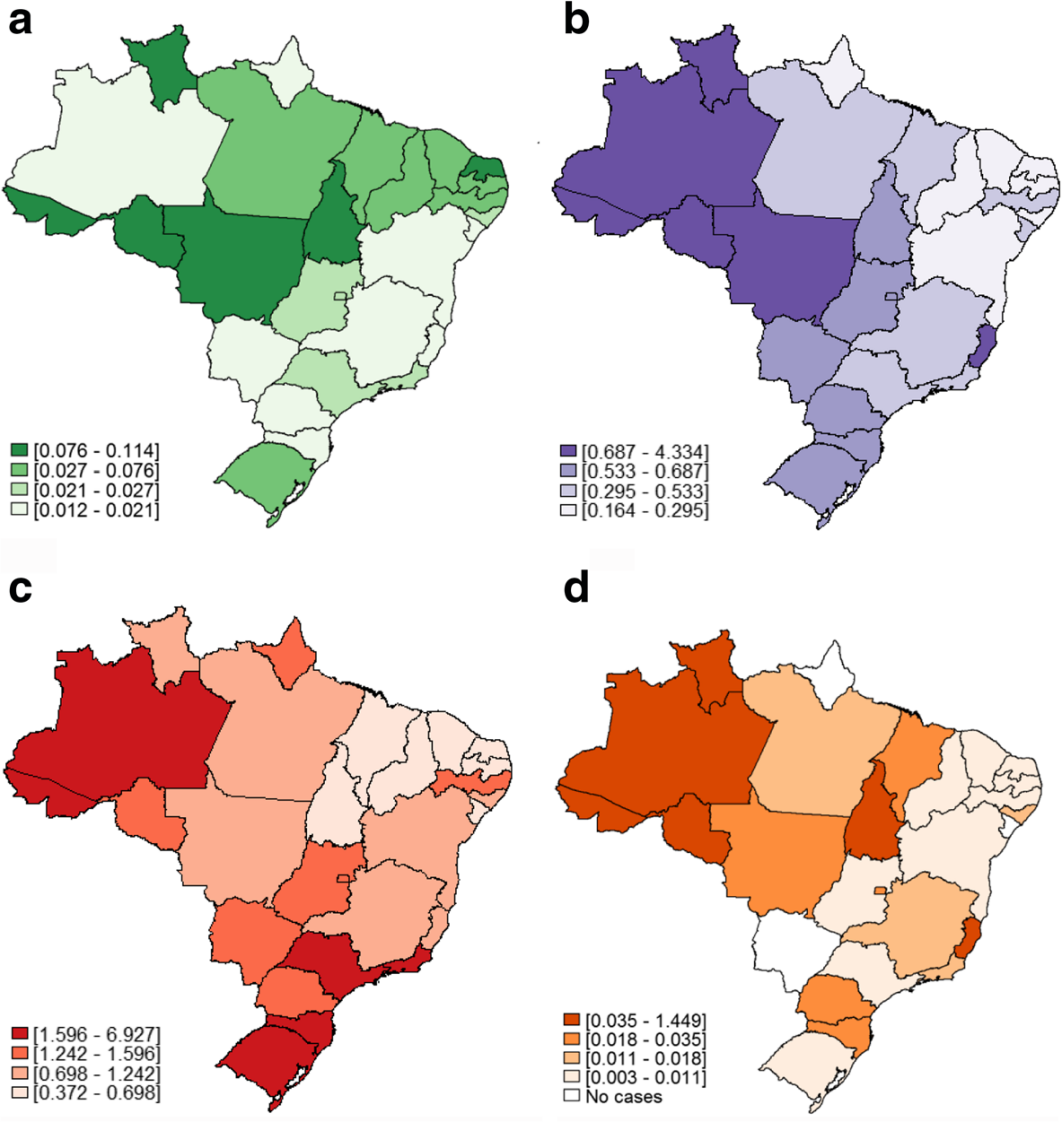
Age-standardized mortality rates (per 100.000 inhabitants) related to: **a** hepatitis A [B15.0; B15.9]; **b** hepatitis B [B16.2; B16.9; B18.1]; **c** hepatitis C [B17.1; B18.2] and **d** hepatitis Delta [B16.0; B16.1; B18.0; B17.0] according to federative units in Brazil from 2008 to 2014. Density of colors expressed in quartiles

The top ranking Brazilian federative units for age-standardized mortality rates [official abbreviation for the state = MR per 100,000 inhabitants (95% CI) related to viral hepatitis were Acre [AC = 12.885 (11.698–14.172)], Rio Grande do Sul [RS = 6.187 (6.023–6.356)] and Amazonas [AM = 4.170 (3.858–4.504)]. On the other hand, Ceará [CE = 0.764 (0.692–0.842)], Piauí [PI = 0.829 (0.705–0.969)] and Paraíba [PB = 0.854 (0.743–0.978)] had the lowest mortality rates associated with viral hepatitis (Table 3 and Fig. 1). As for hepatitis A, B and Delta, the top mortality rates were observed in federative units located in the North region: (i) for hepatitis A: AC = 0.114 (0.034–0.311), Rondônia (RO) = 0.091 (0.037–0.198) and Roraima (RR) = 0.080 (0.016–0.421); (ii) for hepatitis B: AC = 4.334 (3.677–5.089); RO = 1.682 (1.416–1.993) and AM = 1.542 (1.357–1.748); (iii) for hepatitis Delta: AC = 1.449 (1.098–1.895); AM = 0.553 (0.458–0.667) and Roraima (RR) = 0.184 (0.048–0.584). Considering hepatitis C, the highest mortality rates were distributed across the country: AC = 6.927 (6.030–7.929) in North; RS = 5.370 (5.218–5.527) in South and São Paulo (SP) = 3.046 (2.984–3.109) in Southeast region. The northern Brazil’s federative unit called Acre (AC) had the higher mortality rates for hepatitis A, B, C and Delta (Table 4 and Fig. 2). The number of deaths and crude mortality rates due to viral hepatitis A, B, C and Delta according to federate unit in Brazil in the 7-year period of analysis were summarized in the Additional file 1 Table S2. Age-standardized mortality rates due to viral hepatitis by year according to the Brazilian macro-regions were plotted in the Additional file 1: Figure S2.

**Table 3:** Number of deaths, crude and age-standardized mortality rates (per 100,000 inhabitants) [95% confidence interval] due to viral hepatitis according to the federative units in Brazil from 2008 to 2014

Macro-region	Federative Unit	Mean population per year	Number of deaths	Crude mortality rates	Age-standardized mortality rates [95% CI]
North	Acre (AC)	739,518	466	9.002	12.885 [11.698–14.172]
	Amazonas (AM)	3,575,067	737	2.945	4.170 [3.858–4.504]
	Amapa (AP)	682,303	48	1.005	1.926 [1.396–2.609]
	Para (PA)	7,698,641	748	1.388	2.000 [1.855–2.154]
	Rondônia (RO)	1,600,496	310	2.767	3.576 [3.176–4.020]
	Roraima (RR)	457,143	68	2.125	3.282 [2.500–4.280]
	Tocantins (TO)	1,392,251	115	1.180	1.438 [1.183–1.734]
Northeast	Alagoas (AL)	3,191,489	231	1.034	1.308 [1.143–1.491]
	Bahia (BA)	1,4512,661	1215	1.196	1.344 [1.269–1.422]
	Ceara (CE)	8,604,602	415	0.689	0.764 [0.692–0.842]
	Maranhão (MA)	6,609,808	527	1.139	1.487 [1.361–1.621]
	Paraiba (PB)	3,820,185	215	0.804	0.854 [0.743–0.978]
	Pernambuco (PE)	8,948,388	1016	1.622	1.826 [1.715–1.942]
	Piaui (PI)	3,152,709	160	0.725	0.829 [0.705–0.969]
	Rio Grande do Norte (RN)	3,232,637	229	1.012	1.147 [1.003–1.307]
	Sergipe (SE)	2,100,840	145	0.986	1.206 [1.016–1.423]
Southeast	Espirito Santo (ES)	36,15,218	516	2.039	2.153 [1.970–2.349]
	Minas Gerais (MG)	20,050,125	2000	1.425	1.397 [1.336–1.460]
	Rio de Janeiro (RJ)	16,149,068	4082	3.611	3.248 [3.149–3.350]
	São Paulo (SP)	42,116,780	11,548	3.917	3.797 [3.728–3.868]
South	Parana (PR)	10,696,240	1630	2.177	2.163 [2.059–2.271]
	Rio Grande do Sul (RS)	10,906,189	5399	7.072	6.187 [6.023–6.356]
	Santa Catarina (SC)	6,353,717	1252	2.815	2.773 [2.620–2.933]
Central-West	Distrito Federal (DF)	2,661,654	354	1.900	2.349 [2.105–2.616]
	Goias (GO)	6,137,736	803	1.869	2.053 [1.912–2.203]
	Mato Grosso do Sul (MS)	2,476,719	375	2.163	2.347 [2.114–2.600]
	Mato Grosso (MT)	3,084,790	374	1.732	2.023 [1.819–2.247]

Mean population by year = ratio between total population from 2008 to 2014 and 7 years. Crude mortality rate = number of death / mean population * 7 (number of years)

**Table 4:** Age-standardized mortality rates (per 100,000 inhabitants) [95% confidence interval] due to viral hepatitis according to the federative units in Brazil from 2008 to 2014

Macro-region	Federative Unit	Hepatitis A	Hepatitis B	Hepatitis C	Hepatitis Delta
North	Acre (AC)	0.114 [0.034–0.311]	4.334 [3.677–5.089]	6.927 [6.030–7.929]	1.449 [1.098–1.895]
	Amazonas (AM)	0.014 [0.004–0.049**]**	1.542 [1.357–1.748]	1.625 [1.420–1.853]	0.553 [0.458–0.667]
	Amapa (AP)	0.016 [0.001–0.232]	0.273 [0.105–0.610]	1.299 [0.866–1.890]	NA
	Para (PA)	0.071 [0.047–0.105]	0.393 [0.331–0.464]	1.169 [1.056–1.290]	0.018 [0.007–0.039]
	Rondônia (RO)	0.091 [0.037–0.198]	1.682 [1.416–1.993]	1.430 [1.176–1.731]	0.112 [0.052–0.224]
	Roraima (RR)	0.080 [0.016–0.421]	1.323 [0.855–2.013]	1.193 [0.726–1.895]	0.184 [0.048–0.584]
	Tocantins (TO)	0.106 [0.048–0.209]	0.652 [0.484–0.861]	0.372 [0.246–0.542]	0.038 [0.008–0.116]
Northeast	Alagoas (AL)	0.025 [0.008–0.063]	0.294 [0.219–0.387]	0.818 [0.687–0.967]	0.018 [0.004–0.054]
	Bahia (BA)	0.021 [0.013–0.033]	0.242 [0.211–0.277]	0.893 [0.831–0.958]	0.005 [0.002–0.013]
	Ceara (CE)	0.033 [0.021–0.052]	0.180 [0.146–0.219]	0.442 [0.386–0.503]	0.003 [0.001–0.013]
	Maranhão (MA)	0.076 [0.051–0.111]	0.446 [0.379–0.523]	0.652 [0.568–0.745]	0.027 [0.013–0.051]
	Paraiba (PB)	0.051 [0.028–0.087]	0.164 [0.118–0.222]	0.509 [0.423–0.608]	0.004 [0.001–0.024]
	Pernambuco (PE)	0.037 [0.023–0.053]	0.353 [0.306–0.406]	1.308 [1.214–1.408]	0.008 [0.003–0.020]
	Piaui (PI)	0.042 [0.019–0.183]	0.295 [0.224–0.383]	0.375 [0.293–0.475]	0.011 [0.001–0.040]
	Rio Grande do Norte (RN)	0.078 [0.045–0.129]	0.246 [0.182–0.327]	0.698 [0.595–0.826]	0.010 [0.001–0.039]
	Sergipe (SE)	0.014 [0.002–0.059]	0.395 [0.291–0.526]	0.630 [0.493–0.795]	NA
Southeast	Espirito Santo (ES)	0.012 [0.002–0.038]	0.726 [0.622–0.844]	1.242 [1.104–1.394]	0.048 [0.025–0.086]
	Minas Gerais (MG)	0.021 [0.014–0.030]	0.362 [0.332–0.395]	0.881 [0.833–0.931]	0.012 [0.007–0.019]
	Rio de Janeiro (RJ)	0.023 [0.015–0.034]	0.468 [0.431–0.508]	2.605 [2.516–2.696]	0.013 [0.007–0.021]
	São Paulo (SP)	0.027 [0.021–0.033]	0.533 [0.508–0.560]	3.046 [2.984–3.109]	0.011 [0.007–0.015]
South	Parana (PR)	0.016 [0.008–0.028]	0.649 [0.593–0.710]	1.295 [1.215–1.379]	0.020 [0.011–0.033]
	Rio Grande do Sul (RS)	0.030 [0.020–0.046]	0.604 [0.553–0.659]	5.370 [5.218–5.527]	0.011 [0.005–0.022]
	Santa Catarina (SC)	0.021 [0.010–0.042]	0.609 [0.539–0.687]	1.965 [1.837–2.100]	0.033 [0.019–0.054]
Central-West	Distrito Federal (DF)	0.025 [0.006–0.072]	0.687 [0.558–0.840]	1.473 [1.281–1.688]	0.027 [0.007–0.075]
	Goias (GO)	0.024 [0.011–0.046]	0.615 [0.540–0.700]	1.252 [1.142–1.370]	0.011 [0.003–0.028]
	Mato Grosso do Sul (MS)	0.020 [0.004–0.060]	0.589 [0.476–0.722]	1.596 [1.405–1.807]	NA
	Mato Grosso (MT)	0.082 [0.044–0.142]	0.809 [0.682–0.955]	0.916 [0.781–1.072]	0.026 [0.008–0.069]

NA: Not applicable given that no cases were reported. Mean population per year for federative units are described in Table 3

## Discussion

The present study highlighted the age-standardized mortality rates related to viral hepatitis in Brazil in a 7-year period (from 2008 to 2014). Despite relative low age-standardized mortality rates associated with viral hepatitis, Brazil is a large and complex country with nearly 200 million inhabitants and striking regional differences ranging from the richer South and Southeast, the poorer North and Northeast and the expanding Central-West region. This nationwide death registry analysis showed that viral hepatitis were associated with over 34,000 deaths in this period.

A Brazilian study reported that more than 90% of northern and northeast population tested positive for hepatitis A IgG antibodies [16]. This study analyzed of the mortality rates due to hepatitis A in Brazil from 1980 to 2002 reporting higher rates in Northern region and a progressive decrease (from 0.20 to 0.02 per 100,000 inhabitants) in hepatitis A mortality rates in all Brazilian macro-regions [16]. The present study described similar higher mortality rates in North and Northeast compared to South and Southeast regions. However, we described higher age- standardized mortality rates [0.032 (0.029–0.035)] in a 7-years period and a non-significant time trend. These results are alarming particularly because of the inverse correlation between hepatitis A and socioeconomic standards [17]. Regarding hepatitis B, Brazil has been classified as of low endemicity with an estimated prevalence of 0.65% (95% CI 0.65–0.66) [18]. However, prevalence of hepatitis B and Delta has a heterogenic distribution across the country, with higher concentration in Northern Brazil [19]. In the general population, prevalence of HBsAg marker ranged from 0.57% in the Southeast to 6.2% in the North region [20]. More than 60,000 and 70,000 cases of hepatitis B and C, respectively, were registered at the Brazilian National System for Surveillance and Control of Diseases from 2012 to 2015 (http://portalsinan.saude.gov.br/hepatites-virais), respectively. A Brazilian study that analyzed 2936 individuals living in Rio de Janeiro (Southeast region) showed a prevalence of 0.14% (0.01–0.30) hepatitis B and 0.44% (0.20–0.68) hepatitis C infection [21]. However, a higher prevalence of chronic hepatitis C [1.38% (95% CI: 1.12–1.64)] was reported in a second cross-sectional population-based study that analyzed more than 19000 individuals over the country [22]. In addition, using mathematic models, the Brazilian Ministry of Health estimated that around 1,450,000 people are living with hepatitis C [6].

According to the Global Hepatitis Report from the WHO, the global number of deaths related to viral hepatitis increased from 1.10 million deaths in 2000 to 1.34 million deaths in 2015 [23]. In addition, according to the Global Burden Disease (GBD), viral hepatitis has been associated with disability adjusted life year worldwide [24] and the mortality rates (95% CI) from cirrhosis due to hepatitis B and C were 8.1 (5.36–11.49) and 1.7 (0.6–3.4) per 100,000 inhabitants in Brazil in 2013 [25]. Mortality rates from viral hepatitis B and C have increased from 1999 to 2007 in the United States [26]. Moreover, national hospitalization and inpatient mortality rates due to hepatitis C have increased in the last decade [27]. Comparing our data with those from GBD 188 countries [17], age- standardized mortality-rate was lower for hepatitis B and higher for hepatitis C [28]. Our results show that Brazilian mortality rates widely varied according to geographical localization. Hence, southeast and south regions presented higher mortality rates due to hepatitis C than others regions. These results might be explained by higher rates of diagnosis since these populations have a better access to the health care systems [29] and/or by higher incidence of hepatitis C [30]. Higher mortality rates in few Brazilian federations units, such as AC and RS, might be related to a higher prevalence of injection drug users in these regions [31]. Northern Brazil, especially the Amazon Basin, is an endemic area for hepatitis Delta with up to 30% of hepatitis Delta virus infection in patients with hepatitis B [32]. This finding might explain the higher mortality rates due to hepatitis B and Delta in North compared to others Brazilian macro-regions. However, mortality rates comparisons between regions and/or countries should be interpreted with caution due to a considerable variability in death report processes worldwide and in the methods used for data analysis. In the present study, death due to viral hepatitis was defined by the presence of ICD-10 codes in the primary, secondary or contributing causes of death in the DC; mortality rates were age- standardized using the direct method and the standard population proposed by WHO which was estimated as an average world population age-structure constructed for the period 2000–2025. In a sensitivity analysis, we reported similar results on age-standardized mortality rates related to viral hepatitis and a non-significant trend for annual increasing or decreasing mortality rates in all Brazilian macro-regions from 2008 to 2014 when using the Brazilian population from year 2008 as the standard population (Additional file 1: Table S3).

Considering the current age-standardized mortality rates and the population projected by IBGE for the next 50 years, more than 190,000 deaths associated with viral hepatitis might occur from 2020 to 2060 in Brazil, most of them (up to 154,000) due to hepatitis C assuming no improvements in treatment and/or other public health strategies. The Viral Hepatitis National Program from the Brazilian MoH has been prioritizing health policies to improve the cascade of hepatitis B and C, as well as to implement cost-effective prevention programs and novel treatment for viral hepatitis. Accurate point-of-care tests are available in Brazil for hepatitis B screening [33] and vaccination of newborns has been part of the National Immunization Program since 1998. Currently, hepatitis B vaccine is widely available for individuals up to 49 years-old [34]. Vaccination of difficult-to-access populations, such as those living in rural areas or in the Amazon forest, is still a challenge to the prevention of hepatitis B and Delta. Despite the satisfactory vaccine coverage, others prevention and treatment interventions are available, but insufficiently implemented in Brazil. First-line drugs to control hepatitis B and Delta has been delivered free of charge by the Brazilian public health system (*Sistema Único de Saúde* – SUS) [35]. Nucleos(t)ide analogues (NUCs) are available for chronic hepatitis B treatment since 2009. In addition, hepatitis C has been considered as a public health issue by Brazilian health authorities [36]. Despite the absence of a vaccine, point-of-care tests have been used for screening [37] and non-invasive tests have been available for fibrosis staging in Brazilian citizens living with chronic hepatitis C [38]. Improvement in identification of HCV-infected individuals and treatment of those with fibrosis stage F ≥ 2 with highly effective drugs (sustained virological response of 85%) might lead to 90% reduction in hepatitis burden by year 2030 in Brazil [12]. The BMoH had an intense negotiation with pharmaceutical companies resulting in more than 90% discount over international prices for direct-aging antivirals drugs (DAAs), such as sofosbuvir, daclastavir and simeprevir. These DAAs were recently incorporated by the public health system to be delivered for free to the Brazilian population aiming to tackle chronic hepatitis C and to prevent its complications [7, 39]. The surveillance of mortality rates seems to be one of the most important strategies to guide viral hepatitis health policies. The relative short period of free-delivery of NUCs and DAAs by the Brazilian MoH might explain the non-significant decrease in mortality rates in the last decade. However, trends of annual mortality rates can be used in the future to evaluate the efficacy of recently implemented strategies, such as hepatitis C eradication by DAAs.

The present study has several limitations, mainly associated with the absence of standardization by gender and the lack of high quality of death cause registration in few Brazilian regions. However, death count coverage in Brazil has increased from 80% in 1980–1991 to more than 95% in 2000–2010 [40]. In addition, cause-of-death information has highly improved in Brazil after 2006 likely resulting from investments in public health care system and death report process in this country [41]. Accordingly, we analyzed mortality data from 2008 onwards to avoid the use and interpretation of low-quality data. The proportion of deaths from ill-defined or unknown causes of death [ICD-10: R00-R99] might be used as an indicator of the quality of cause-of-death data [42]. Quality of mortality data seems to be improving in the last years in Brazil: from 2000 to 2012, the proportion of ill-defined or unknown causes of death has decreased from 14.3–6.3% of deaths [11]. In addition, the number of ill-defined deaths and the mortality rates of unknown cause of deaths have decreased by 45% and 52%, respectively [11]. These decrease in proportion, absolute number of deaths and mortality rates were observed in all Brazilian macro-regions. However, there are still large regional differences in mortality report in Brazil: data quality seems to be better in South and Southeast compared to others regions [43]. Gender differences in mortality and life expectancy vary by country. However, in most countries, mortality rates are higher in men compared to women. Analysis of mortality rates with sex-standardization would be important for implementation of different health strategies in male and female population.

The major strength of this study was the estimation of the burden of viral hepatitis mortality analyzing individual data in a nationwide database (SIM) provided by the Brazilian MoH. SIM registers death causes based on DC classified by ICD codes since 1979 (ICD-9 from 1979 to 1995 and ICD-10 from 1996 onwards). Since 1999, primary, secondary and contributing causes of death have been available in the database. Mortality data has been systematically updated by the Brazilian Unified Health System Information Technology Department. Death by viral hepatitis was defined as presence of the specific ICD-10 in any field of the DC to avoid underestimation of mortality rates. In addition, mortality rates were adjusted for age using the WHO world standard population that allows comparison of our data with different countries worldwide. Moreover, the consideration of Brazil as a whole as well as its macro-regions can contribute to a greater understanding of the burden of liver disease in the country and perhaps worldwide.

## Conclusion

In summary, this study described the age- standardized mortality rates associated with viral hepatitis in Brazil and its micro and macro-regions from 2008 to 2012. Viral hepatitis were associated with up to 22,000 deaths, most of them related to hepatitis C. Mortality rates were not homogeneous across the country, suggesting that health policies, prevention programs and novel treatments should be tailored in Brazil according to geographical location.

## Additional file

Additional file 1: **Supplementary materials: Table S1.** Age-standardized mortality rates (aMR) and annual percent change (%∆) after age-standardization from mortality rates related to viral hepatitis according to Brazilian macro-regions. **Table S2.** Number of deaths and crude mortality rates (per 100,000 inhabitants) related to hepatitis A, B, C and Delta according to the federative units in Brazil from 2008 to 2014. **Table S3.** Age-standardized mortality rates (per 100.000 inhabitants) [95% confidence interval] due to viral hepatitis according to the macro-regions and federative units in Brazil from 2008 to 2014 using the Brazilian population from year 2008 as the standard population. **Figure S1.** Graphical representation of population distribution in the federative units of Brazil. Population expressed as mean of millions inhabitants per year from 2008 to 2014. **Figure S2.** Age-standardized mortality rates (per 100.000 inhabitants) related to viral hepatitis by year according to the Brazilian macro-regions. (DOCX 1674 kb)

## Abbreviations

CI: Confidence interval
DAA: Direct acting agents
DATASUS: Brazilian Unified Health System Information Technology Department
DC: Death certificate
EU: European Union
IBGE: Brazilian Institute of Geography and Statistics
ICD: International Classification of Diseases
MoH: Ministry of Health
NUCs: Nucleos(t)ide analogues
SIM: Sistema de Informação sobre Mortalidade - National Registry of Death database
SINAN: Sistema de Informação de Agravos de Notificação
SUS: Sistema Único de Saúde
WHO: World Health Organization
