# Supplementary material for: Age-standardized mortality rates related to viral hepatitis in Brazil
Source: BMC Infect Dis. 2017 Jul 31;17:527. doi: 10.1186/s12879-017-2619-y (PMC5537933; doi:10.1186/s12879-017-2619-y)

**Supplementary Material**

**Age-standardized mortality rates related to viral hepatitis in Brazil**

**Author Names**:

Hugo Perazzo ^1^, Antonio G Pacheco ², Paula M Luz ^1^, Rodolfo Castro ^1^, Chris Hyde ^3^, Juliana Fittipaldi ^1^, Caroline Rigolon ^1^, Sandra W Cardoso^1^, Beatriz Grinsztejn ^1^, Valdiléa G Veloso ^1^

| **Supplementary Tables** | **Page** |
| --- | --- |
| **Additional file 1: Table S1.** Age-standardized mortality rates (aMR) and annual percent change (%∆) after age-standardization from mortality rates related to viral hepatitis according to Brazilian macro-regions | 1 |
| **Additional file 1: Table S2.** Number of deaths and crude mortality rates (per 100,000 inhabitants) related to hepatitis A, B, C and Delta according to the federative units in Brazil from 2008 to 2014 | 2 |
| **Additional file 1: Table S3.** Age-standardized mortality rates (per 100.000 inhabitants) [95% confidence interval] due to viral hepatitis according to the macro-regions and federative units in Brazil from 2008 to 2014 using the Brazilian population from year 2008 as the standard population | 3 |
| **Supplementary Figures** |  |
| **Additional file 1: Figure S1.** Graphical representation of population distribution in the federative units of Brazil. Population expressed as mean of millions inhabitants per year from 2008 to 2014. | 4 |
| **Additional file 1: Figure S2.** Age-standardized mortality rates (per 100.000 inhabitants) related to viral hepatitis by year according to the Brazilian macro-regions | 5 |

**Additional file 1: Table S1.** Age-standardized mortality rates (aMR) and annual percent change (**%∆)** after age-standardization from mortality rates related to viral hepatitis according to Brazilian macro-regions

|  | **aMR N** | **Annual**  **%∆** | **aMR**  **NE** | **Annual**  **%∆** | **aMR**  **SE** | **Annual**  **%∆** | **aMR**  **S** | **Annual**  **%∆** | **aMR CW** | **Annual**  **%∆** | **aMR**  **BRAZIL** | **Annual**  **%∆** |
| --- | --- | --- | --- | --- | --- | --- | --- | --- | --- | --- | --- | --- |
| **Hepatitis A [B15.0; B15.9]** | | | | | | | | | | | | |
| 2008 | 0.129 | - | 0.044 | - | 0.038 | - | 0.019 | - | 0.090 | - | 0.045 | - |
| 2009 | 0.086 | -33.3 | 0.047 | 6.8 | 0.034 | -10.5 | 0.047 | 147.4 | 0.046 | -48.9 | 0.043 | -4.4 |
| 2010 | 0.094 | 9.3 | 0.048 | 2.1 | 0.016 | -52.9 | 0.033 | -29.8 | 0.016 | -65.2 | 0.033 | -23.3 |
| 2011 | 0.045 | -52.1 | 0.023 | -52.1 | 0.028 | 75.0 | 0.014 | -57.6 | 0.009 | -43.8 | 0.025 | -24.2 |
| 2012 | 0.034 | -24.4 | 0.042 | 82.6 | 0.022 | -21.4 | 0.028 | 100.0 | 0.054 | 500.0 | 0.031 | 24.0 |
| 2013 | 0.071 | 108.8 | 0.035 | -16.7 | 0.014 | -36.4 | 0.013 | -53.6 | 0.013 | -75.9 | 0.025 | -19.4 |
| 2014 | 0.023 | -67.6 | 0.036 | 2.9 | 0.017 | 21.4 | 0.009 | -30.8 | 0.026 | 100.0 | 0.022 | -12.0 |
| **Hepatitis B [B16.2; B16.9; B18.1]** | | | | | | | | | | | | |
| 2008 | 1.247 | - | 0.277 | - | 0.525 | - | 0.707 | - | 0.704 | - | 0.551 | - |
| 2009 | 0.893 | -28.4 | 0.286 | 3.2 | 0.520 | -1.0 | 0.625 | -11.6 | 0.733 | 4.1 | 0.518 | -6.0 |
| 2010 | 0.938 | 5.0 | 0.264 | -7.7 | 0.573 | 10.2 | 0.657 | 5.1 | 0.701 | -4.4 | 0.541 | 4.4 |
| 2011 | 1.092 | 16.4 | 0.292 | 10.6 | 0.543 | -5.2 | 0.693 | 5.5 | 0.553 | -21.1 | 0.539 | -0.4 |
| 2012 | 0.904 | -17.2 | 0.282 | -3.4 | 0.46 | -15.3 | 0.544 | -21.5 | 0.608 | 9.9 | 0.467 | -13.4 |
| 2013 | 0.987 | 9.2 | 0.264 | -6.4 | 0.387 | -15.9 | 0.606 | 11.4 | 0.709 | 16.6 | 0.451 | -3.4 |
| 2014 | 0.981 | -0.6 | 0.296 | 12.1 | 0.407 | 5.2 | 0.531 | -12.4 | 0.631 | -11.0 | 0.453 | 0.4 |
| **Hepatitis C [B17.1; B18.2]** | | | | | | | | | | | | |
| 2008 | 1.070 | - | 0.772 | - | 2.436 | - | 3.044 | - | 1.207 | - | 1.960 | - |
| 2009 | 1.309 | 22.3 | 0.768 | -0.5 | 2.366 | -2.9 | 3.057 | 0.4 | 1.324 | 9.7 | 1.950 | -0.5 |
| 2010 | 1.314 | 0.4 | 0.716 | -6.8 | 2.447 | 3.4 | 3.051 | -0.2 | 0.952 | -28.1 | 1.935 | -0.8 |
| 2011 | 1.721 | 31.0 | 0.786 | 9.8 | 2.391 | -2.3 | 3.191 | 4.6 | 1.397 | 46.7 | 2.002 | 3.5 |
| 2012 | 1.766 | 2.6 | 0.891 | 13.4 | 2.331 | -2.5 | 3.554 | 11.4 | 1.416 | 1.4 | 2.063 | 3.0 |
| 2013 | 1.413 | -20.0 | 0.795 | -10.8 | 2.262 | -3.0 | 3.069 | -13.6 | 1.291 | -8.8 | 1.897 | -8.0 |
| 2014 | 1.621 | 14.7 | 0.758 | -4.7 | 2.337 | 3.3 | 3.137 | 2.2 | 1.368 | 6.0 | 1.952 | 2.9 |
| **Hepatitis Delta [B16.0; B16.1; B18.0; B17.0]** | | | | | | | | | | | | |
| 2008 | 0.216 | - | 0.003 | - | 0.023 | - | 0.029 | - | 0.041 | - | 0.033 | - |
| 2009 | 0.150 | -30.6 | 0.014 | 366.7 | 0.013 | -43.5 | 0.01 | -65.5 | 0.001 | -97.6 | 0.022 | -33.3 |
| 2010 | 0.156 | 4.0 | 0.009 | -35.7 | 0.019 | 46.2 | 0.014 | 40.0 | NA | NA | 0.027 | 22.7 |
| 2011 | 0.243 | 55.8 | 0.019 | 111.1 | 0.012 | -36.8 | 0.017 | 21.4 | 0.019 | - | 0.031 | 14.8 |
| 2012 | 0.201 | -17.3 | 0.002 | -89.5 | 0.011 | -8.3 | 0.037 | 117.6 | 0.009 | -52.6 | 0.027 | -12.9 |
| 2013 | 0.274 | 36.3 | 0.006 | 200.0 | 0.005 | -54.5 | 0.022 | -40.5 | 0.018 | 100.0 | 0.027 | 0.0 |
| 2014 | 0.295 | 7.7 | 0.012 | 100.0 | 0.010 | 100.0 | 0.007 | -68.2 | 0.010 | -44.4 | 0.03 | 11.1 |
| **All viral hepatitis [B15.0; B15.9; B16.2; B16.9; B18.1; B17.1; B18.2; B16.0; B16.1; B18.0; B17.0; B17.2; B17.8; B18.8; B18.9; B19.0; B19.9]** | | | | | | | | | | | | |
| 2008 | 3.116 | - | 1.266 | - | 3.177 | - | 3.898 | - | 2.253 | - | 2.764 | - |
| 2009 | 2.837 | -9.0 | 1.277 | 0.9 | 3.095 | -2.6 | 3.933 | 0.9 | 2.260 | 0.3 | 2.717 | -1.7 |
| 2010 | 2.816 | -0.7 | 1.211 | -5.2 | 3.219 | 4.0 | 3.923 | -0.3 | 1.810 | -19.9 | 2.712 | -0.2 |
| 2011 | 3.398 | 20.7 | 1.273 | 5.1 | 3.163 | -1.7 | 4.05 | 3.2 | 2.203 | 21.7 | 2.784 | 2.7 |
| 2012 | 3.196 | -5.9 | 1.361 | 6.9 | 2.966 | -6.2 | 4.347 | 7.3 | 2.22 | 0.8 | 2.748 | -1.3 |
| 2013 | 3.017 | -5.6 | 1.235 | -9.3 | 2.822 | -4.9 | 3.93 | -9.6 | 2.153 | -3.0 | 2.565 | -6.7 |
| 2014 | 3.243 | 7.5 | 1.255 | 1.6 | 2.896 | 2.6 | 3.864 | -1.7 | 2.162 | 0.4 | 2.612 | 1.8 |

aMR. age-standardized mortality rate; %∆. annual percent change; N. North; NE. Northeast; SE. Southeast; S. South; CW. Center-West. NA: Not applicable given that no cases were reported. Mean population per year (in millions inhabitants): North =16.145; Northeast=54.173; Southeast=81.931; South=27.956; Central-West=14.360 and Brazil=194.567.

**Additional file 1: Table S2.** Number of deaths and crude mortality rates (per 100,000 inhabitants) related to hepatitis A, B, C and Delta according to the federative units in Brazil from 2008 to 2014

|  |  | **Hepatitis A** | | **Hepatitis B** | | **Hepatitis C** | | **Hepatitis Delta** | |
| --- | --- | --- | --- | --- | --- | --- | --- | --- | --- |
|  | **Federative Unit** | **Deaths**  **(n)** | **Mortality rates** | **Deaths**  **(n)** | **Mortality rates** | **Deaths**  **(n)** | **Mortality rates** | **Deaths**  **(n)** | **Mortality rates** |
| **North** | **Acre (AC)** | 5 | 0.097 | 170 | 3.284 | 122 | 4.289 | 62 | 1.198 |
|  | **Amazonas (AM)** | 4 | 0.016 | 281 | 1.123 | 242 | 0.967 | 126 | 0.503 |
|  | **Amapa (AP)** | 1 | 0.021 | 8 | 0.167 | 30 | 0.628 | 0 | NA |
|  | **Para (PA)** | 29 | 0.054 | 151 | 0.280 | 410 | 0.761 | 7 | 0.013 |
|  | **Rondônia (RO)** | 8 | 0.071 | 151 | 1.348 | 117 | 1.044 | 10 | 0.089 |
|  | **Roraima (RR)** | 3 | 0.094 | 29 | 0.906 | 22 | 0.688 | 4 | 0.126 |
|  | **Tocantins (TO)** | 9 | 0.092 | 52 | 0.533 | 28 | 0.287 | 3 | 0.031 |
| **Northeast** | **Alagoas (AL)** | 5 | 0.022 | 53 | 0.237 | 139 | 0.622 | 3 | 0.013 |
|  | **Bahia (BA)** | 21 | 0.021 | 222 | 0.219 | 790 | 0.778 | 5 | 0.005 |
|  | **Ceara (CE)** | 21 | 0.035 | 98 | 0.163 | 232 | 0.385 | 2 | 0.003 |
|  | **Maranhão (MA)** | 31 | 0.067 | 160 | 0.346 | 221 | 0.478 | 10 | 0.022 |
|  | **Paraiba (PB)** | 14 | 0.052 | 42 | 0.157 | 124 | 0.464 | 1 | 0.004 |
|  | **Pernambuco (PE)** | 22 | 0.035 | 199 | 0.318 | 720 | 1.150 | 5 | 0.008 |
|  | **Piaui (PI)** | 9 | 0.041 | 58 | 0.263 | 70 | 0.317 | 2 | 0.009 |
|  | **Rio Grande do Norte (RN)** | 16 | 0.071 | 49 | 0.217 | 137 | 0.606 | 2 | 0.009 |
|  | **Sergipe (SE)** | 2 | 0.014 | 49 | 0.333 | 73 | 0.496 | 0 | NA |
| **Southeast** | **Espirito Santo (ES)** | 3 | 0.012 | 175 | 0.692 | 297 | 1.174 | 12 | 0.047 |
|  | **Minas Gerais (MG)** | 30 | 0.021 | 521 | 0.371 | 1260 | 0.897 | 17 | 0.012 |
|  | **Rio de Janeiro (RJ)** | 28 | 0.025 | 584 | 0.517 | 3283 | 2.904 | 16 | 0.014 |
|  | **São Paulo (SP)** | 78 | 0.026 | 1620 | 0.549 | 9271 | 3.144 | 33 | 0.011 |
| **South** | **Parana (PR)** | 12 | 0.016 | 489 | 0.653 | 978 | 1.306 | 15 | 0.020 |
|  | **Rio Grande do Sul (RS)** | 25 | 0.033 | 523 | 0.685 | 4693 | 6.148 | 10 | 0.013 |
|  | **Santa Catarina (SC)** | 9 | 0.020 | 273 | 0.614 | 893 | 2.008 | 15 | 0.034 |
| **Central-West** | **Distrito Federal (DF)** | 4 | 0.021 | 103 | 0.553 | 221 | 1.186 | 4 | 0.0421 |
|  | **Goias (GO)** | 9 | 0.021 | 243 | 0.566 | 487 | 1.133 | 4 | 0.009 |
|  | **Mato Grosso do Sul (MS)** | 3 | 0.017 | 95 | 0.548 | 255 | 1.471 | 0 | NA |
|  | **Mato Grosso (MT)** | 15 | 0.069 | 151 | 0.699 | 168 | 0.778 | 5 | 0.023 |

NA: Not applicable given no cases were reported. Mean population per year for federative units as described in Table 3.

**Additional file 1: Table S3.** Age-standardized mortality rates (per 100.000 inhabitants) [95% confidence interval] due to viral hepatitis according to the macro-regions and federative units in Brazil from 2008 to 2014 using the Brazilian population from year 2008 as the standard population

|  | **Hepatitis A** | **Hepatitis B** | **Hepatitis C** | **Hepatitis Delta** | **All** |
| --- | --- | --- | --- | --- | --- |
| **North** | **0.061 [0.046-0.080]** | **0.906 [0.845-0.971]** | **1.272 [1.196-1.351]** | **0.217 [0.188-0.249]** | **2.766 [2.657-2.879]** |
| **Acre (AC)** | 0.111 [0.034-0.287] | 4.047 [3.447-4.730] | 6.056 [3.447-4.730] | 1.409 [1.074-1.828] | 11.707[10.648-12.851] |
| **Amazonas (AM)** | 0.014 [0.004-0.047] | 1.434 [1.267-1.621] | 1.413 [1.238-1.609] | 0.558 [0.463-0.669] | 3.828 [3.549-4.126] |
| **Amapa (AP)** | 0.016 [0.001-0.196] | 0.239 [0.095-0.523] | 1.116 [0.748-1.617] | NA | 1.662 [1.211-2.241] |
| **Para (PA)** | 0.065 [0.043-0.096] | 0.351 [0.297-0.413] | 1.016 [0.919-1.121] | 0.016 [0.006-0.035] | 1.774 [1.648-1.909] |
| **Rondônia (RO)** | 0.083 [0.035-0.180] | 1.533 [1.294-1.812] | 1.257 [1.035-1.519] | 0.101 [0.048-0.202] | 3.219 [2.864-3.614] |
| **Roraima (RR)** | 0.081 [0.016-0.387] | 1.217 [0.798-1.831] | 1.056 [0.650-1.668] | 0.167 [0.045-0.522] | 2.970 [2.278-3.848] |
| **Tocantins (TO)** | 0.099 [0.045-0.193] | 0.589 [0.439-0.776] | 0.328 [0.218-0.477] | 0.036 [0.007-0.109] | 1.307 [1.078-1.573] |
| **Northeast** | **0.037 [0.031-0.044]** | **0.252 [0.236-0.269]** | **0.683 [0.656-0.710]** | **0.008 [0.006-0.012]** | **1.126 [1.092-1.161]** |
| **Alagoas (AL)** | 0.023 [0.007-0.055] | 0.265 [0.198-0.347] | 0.705 [0.592-0.833] | 0.016 [0.003-0.047] | 1.154 [1.009-1.314] |
| **Bahia (BA)** | 0.020 [0.013-0.031] | 0.220 [0.192-0.252] | 0.791 [0.737-0.848] | 0.005 [0.002-0.012] | 1.210 [1.143-1.281] |
| **Ceara (CE)** | 0.033 [0.020-0.051] | 0.164 [0.133-0.200] | 0.391 [0.342-0.445] | 0.003 [0.001-0.012] | 0.692 [0.627-0.762] |
| **Maranhão (MA)** | 0.071 [0.048-0.103] | 0.407 [0.346-0.475] | 0.571 [0.498-0.652] | 0.025 [0.012-0.046] | 1.333 [1.221-1.453] |
| **Paraiba (PB)** | 0.047 [0.025-0.080] | 0.154 [0.111-0.209] | 0.452 [0.375-0.539] | 0.003 [0.001-0.021] | 0.776 [0.675-0.888] |
| **Pernambuco (PE)** | 0.035 [0.022-0.053] | 0.315 [0.273-0.363] | 1.127 [1.046-1.212] | 0.008 [0.002-0.018] | 1.598 [1.201-1.699] |
| **Piaui (PI)** | 0.040 [0.018-0.078] | 0.270 [0.205-0.349] | 0.329 [0.256-0.417] | 0.009 [0.001-0.035] | 0.744 [0.633-0.870] |
| **Rio Grande do Norte (RN)** | 0.070 [0.040-0.115] | 0.219 [0.162-0.290] | 0.608 [0.510-0.719] | 0.009 [0.001-0.033] | 1.014 [0.887-1.155] |
| **Sergipe (SE)** | 0.014 [0.002-0.054] | 0.360 [0.266-0.477] | 0.547 [0.429-0.689] | NA | 1.073 [0.905-1.264] |
| **Southeast** | **0.022 [0.018-0.026]** | **0.437 [0.421-0.454]** | **2.095 [2.060-2.130]** | **0.012 [0.009-0.015]** | **2.709 2.669-2.749]** |
| **Espirito Santo (ES)** | 0.012 [0.002-0.036] | 0.646 [0.553-0.750] | 1.088 [0.968-1.220] | 0.044 [0.023-0.079] | 1.899 [1.739-2.072] |
| **Minas Gerais (MG)** | 0.019 [0.013-0.028] | 0.329 [0.301-0.359] | 0.786 [0.743-0.831] | 0.011 [0.006-0.017] | 1.256 [1.201-1.313] |
| **Rio de Janeiro (RJ)** | 0.021 [0.014-0.031] | 0.423 [0.389-0.459] | 2.268 [2.191-2.347] | 0.012 [0.007-0.020] | 2.852 [2.765-2.942] |
| **São Paulo (SP)** | 0.024 [0.019-0.030] | 0.479 [0.456-0.503] | 2.708 [2.654-2.764] | 0.010 [0.007-0.014] | 3.388 [3.326-3.450] |
| **South** | **0.021 [0.015-0.028]** | **0.558 [0.528-0.590]** | **2.826 [2.757-2.895]** | **0.017 [0.012-0.024]** | **3.575 [3.498-3.654]** |
| **Parana (PR)** | 0.014 [0.007-0.026] | 0.580 [0.530-0.634] | 1.156 [1.084-1.231] | 0.018 [0.010-0.030] | 1.933 [1.840-2.030] |
| **Rio Grande do Sul (RS)** | 0.028 [0.018-0.043] | 0.543 [0.497-0.593] | 4.800 [4.662-4.941] | 0.010 [0.005-0.020] | 5.536 [5.388-5.688] |
| **Santa Catarina (SC)** | 0.020 [0.009-0.039] | 0.551 [0.487-0.621] | 1.779 [1.664-1.901] | 0.030 [0.017-0.050] | 2.508 [2.370-2.652] |
| **Central-West** | **0.033 [0.022-0.047]** | **0.599 [0.552-0.650]** | **1.149 1.082-1.218]** | **0.016 0.009-0.029]** | **1.938 [1.852-2.028]** |
| **Distrito Federal (DF)** | 0.025 [0.007-0.068] | 0.615 [0.501-0.750] | 1.310 [1.141-1.499] | 0.023 [0.006-0.065] | 2.100 [1.885-2.336] |
| **Goias (GO)** | 0.021 [0.010-0.042] | 0.560 [0.492-0.636] | 1.123 [1.025-1.228] | 0.010 [0.009-0.027] | 1.855 [1.728-1.989] |
| **Mato Grosso do Sul (MS)** | 0.019 [0.004-0.055] | 0.533 [0.431-0.652] | 1.427 [1.257-1.615] | NA | 2.110 [1.901-2.336] |
| **Mato Grosso (MT)** | 0.078 [0.043-0.133] | 0.735 [0.621-0.866] | 0.821 [0.701-0.960] | 0.024 [0.008-0.063] | 1.833 [1.650-2.034] |
| **Brazil** | **0.030 [0.027-0.033]** | **0.453 [0.442-0.464]** | **1.738 [1.717-1.760]** | **0.027 [0.024-0.030]** | **2.406 [2.381-2.431]** |

NA: Not applicable given that no cases were reported

**Additional file 1: Figure S1.** Graphical representation of the population distribution in the federative units of Brazil. Population expressed as mean of millions inhabitants per year from 2008 to 2014.

**Additional file 1: Figure S2**. Age-standardized mortality rates (per 100.000 inhabitants) related to viral hepatitis by year according to the Brazilian macro-regions


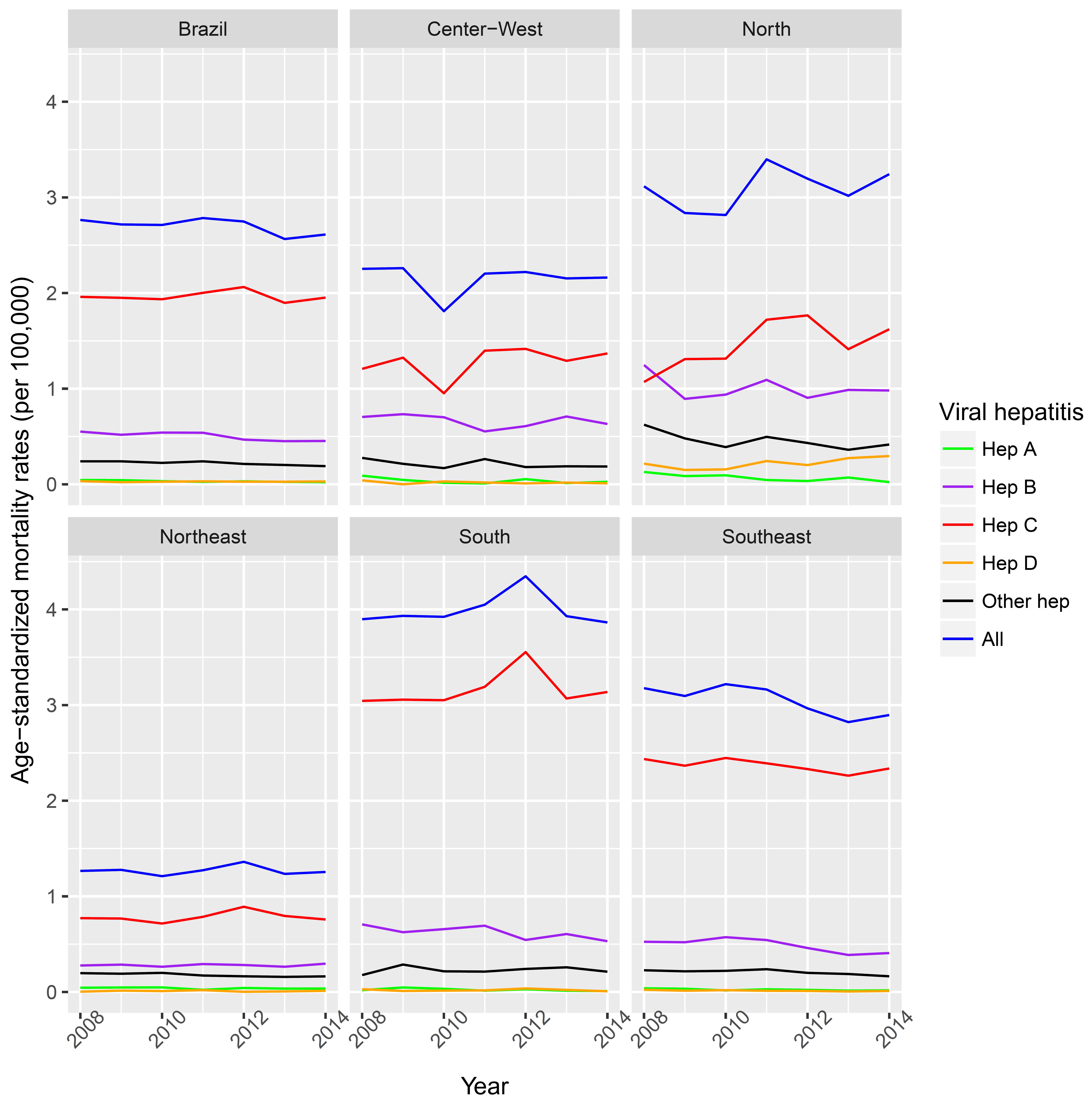

Supplement: Supplementary file 1 — Supplementary materials: Table S1. Age-standardized mortality rates (aMR) and annual percent change (%∆) after age-standardization from mortality rates related to viral hepatitis according to Brazilian macro-regions. Table S2. Number of deaths and crude mortality rates (per 100,000 inhabitants) related to hepatitis A, B, C and Delta according to the federative units in Brazil from 2008 to 2014. Table S3. Age-standardized mortality rates (per 100.000 inhabitants) [95% confidence interval] due to viral hepatitis according to the macro-regions and federative units in Brazil from 2008 to 2014 using the Brazilian population from year 2008 as the standard population. Figure S1. Graphical representation of population distribution in the federative units of Brazil. Population expressed as mean of millions inhabitants per year from 2008 to 2014. Figure S2. Age-standardized mortality rates (per 100.000 inhabitants) related to viral hepatitis by year according to the Brazilian macro-regions. (DOCX 1674 kb) [file 12879_2017_2619_MOESM1_ESM.docx]
